# Supplementary material for: Magnolol and Luteolin Inhibition of α-Glucosidase Activity: Kinetics and Type of Interaction Detected by In Vitro and In Silico Studies
Source: Pharmaceuticals (Basel). 2022 Feb 8;15(2):205. doi: 10.3390/ph15020205 (PMC8880268; doi:10.3390/ph15020205)
Supplement: Supplementary file 1 [file pharmaceuticals-15-00205-s001.zip › pharmaceuticals-1553189-supplementary.pdf]

pharmaceuticals

Article

Magnolol and luteolin inhibition of α-glucosidase activity: kinetics and type of interaction detected by in vitro and in silico studies

Francine Medjiofack Djeujo[1], Eugenio Ragazzi[1], Miriana Urettini[1], Beatrice Sauro[1], Elena Cichero[2]*, Michele Tonelli[2] and Guglielmina Froldi[1]*

[1] Department of Pharmaceutical and Pharmacological Sciences, University of Padova, Padova, Italy; francine.medjiofackdjeujo@phd.unipd.it (FMD), eugenio.ragazzi@unipd.it (ER)
[2] Department of Pharmacy, University of Genova, Genova, Italy; tonelli@difar.unige.it (MT)

* Correspondence: g.froldi@unipd.it; Tel.: +39-049-827-5092; Fax: +39-049-827-5093 (GF); cichero@difar.unige.it (EC)

Citation: Djeujo, F.M.; Ragazzi, E.; Urettini, M.; Sauro, B.; Cichero, E.; Tonelli, M.; Froldi, G. Magnolol and Luteolin Inhibition of α-Glucosidase Activity: Kinetics and Type of Interaction Detected by In Vitro and In Silico Studies. *Pharmaceuticals* **2022**, *15*, 205. https://doi.org/10.3390/ph15020205

Academic Editors: Víctor López and Filippo Maggi

Received: 28 December 2021
Accepted: 4 February 2022
Published: 8 February 2022

Publisher's Note: MDPI stays neutral with regard to jurisdictional claims in published maps and institutional affiliations.

Copyright: © 2022 by the authors. Licensee MDPI, Basel, Switzerland. This article is an open access article distributed under the terms and conditions of the Creative Commons Attribution (CC BY) license (https://creativecommons.org/licenses/by/4.0/).

Supplementary materials

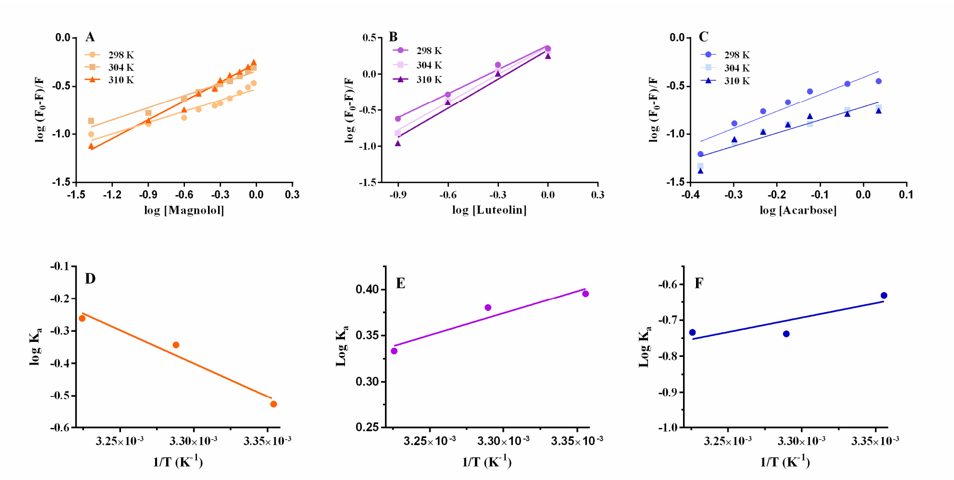

Figure S1 Quenching effect of magnolol (**A**), luteolin (**B**) and acarbose (**C**) on 0.35 μM α-glucosidase fluorescence at three different temperatures (298, 304 and 310 K). Van't Hoff plots for the interaction between α-glucosidase and magnolol (**D**), luteolin (**E**) and acarbose (**F**).

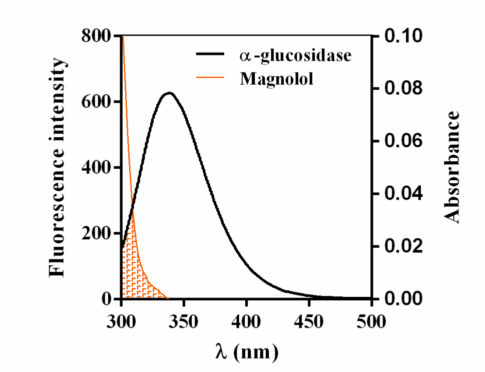

Figure S2 Spectral overlaps of the fluorescence spectrum of α-glucosidase with the UV absorption spectrum of magnolol. Spectra were collected in the wavelength range of 300–500 nm, at room temperature (pH 6.8) and at a concentration of 0.35 μM.

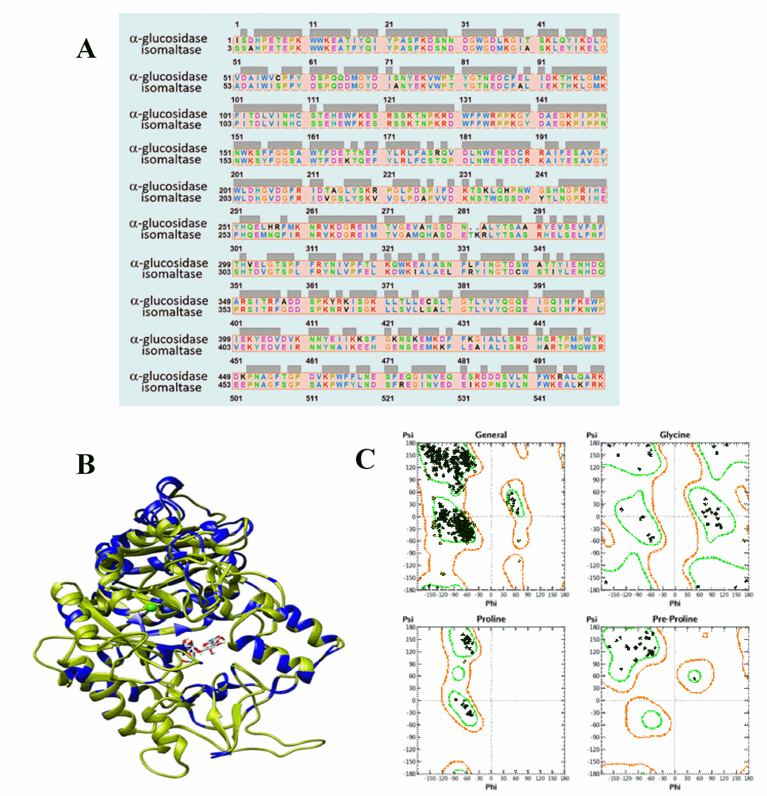

Figure S3 Alignment of the target protein α-glucosidase with respect to template isomaltase. The conserved regions are highlighted by gray stripes (**A**). Superimposition of the modeled α-glucosidase with the X-ray data of the isomaltase. The conserved regions are shown in green. The positioning of isomaltase within the two aforementioned enzymes is shown in cyan and white (**B**). The Ramachandran plot describes the stereochemical quality of the modeled protein. The plot combines four separate Ramachandran maps to distinguish the membership to a particular class (general, proline, glycine, and pre-proline). The colour scheme encodes the affiliation to a certain region within the respective Ramachandran map. Residues in the core region are rendered green or yellow if they are found to be in the allowed region and red if they are in the outlier region. Outliers are always marked as crosses, independent of their class (**C**).

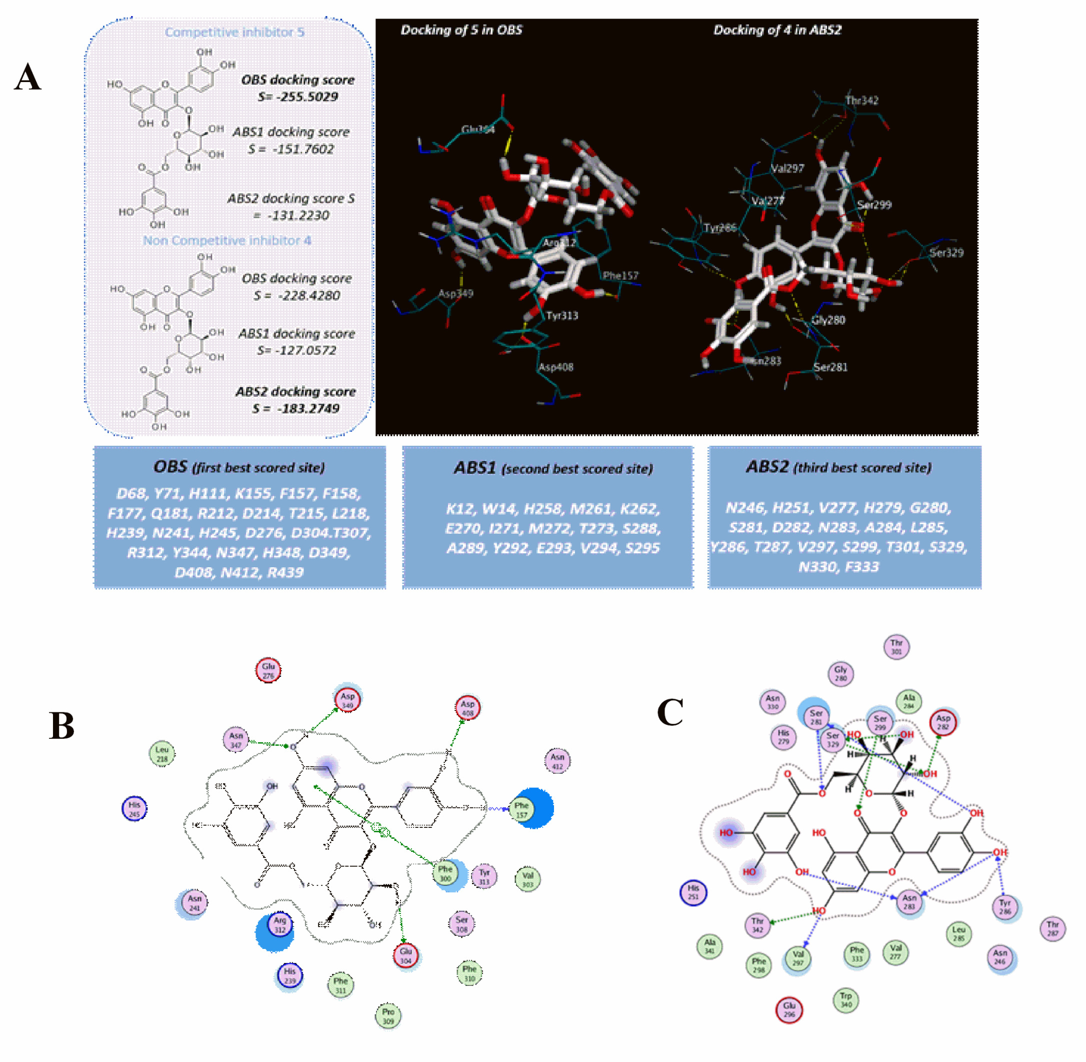

Figure S4 Docking position of α-glucosidase inhibitors **5** and **4** at the OBS and ABS2 binding sites (**A**). Only the most relevant residues are depicted. The amino acids included in OBS, ABS1, and ABS2 are listed. Ligplot B reports the most relevant contacts between α-glucosidase and the competitive inhibitor **5**. Hydrophobic and polar residues are shown in green and pink, respectively, with negative and positive charged amino acids detailed by red and blue circles (**B**). Ligplot C reports the most relevant contacts between α-glucosidase and the non-competitive inhibitor **4**. Hydrophobic and polar residues are shown in green and pink, respectively, being the negative and positive-charged amino acids detailed by red and blue circles (**C**).

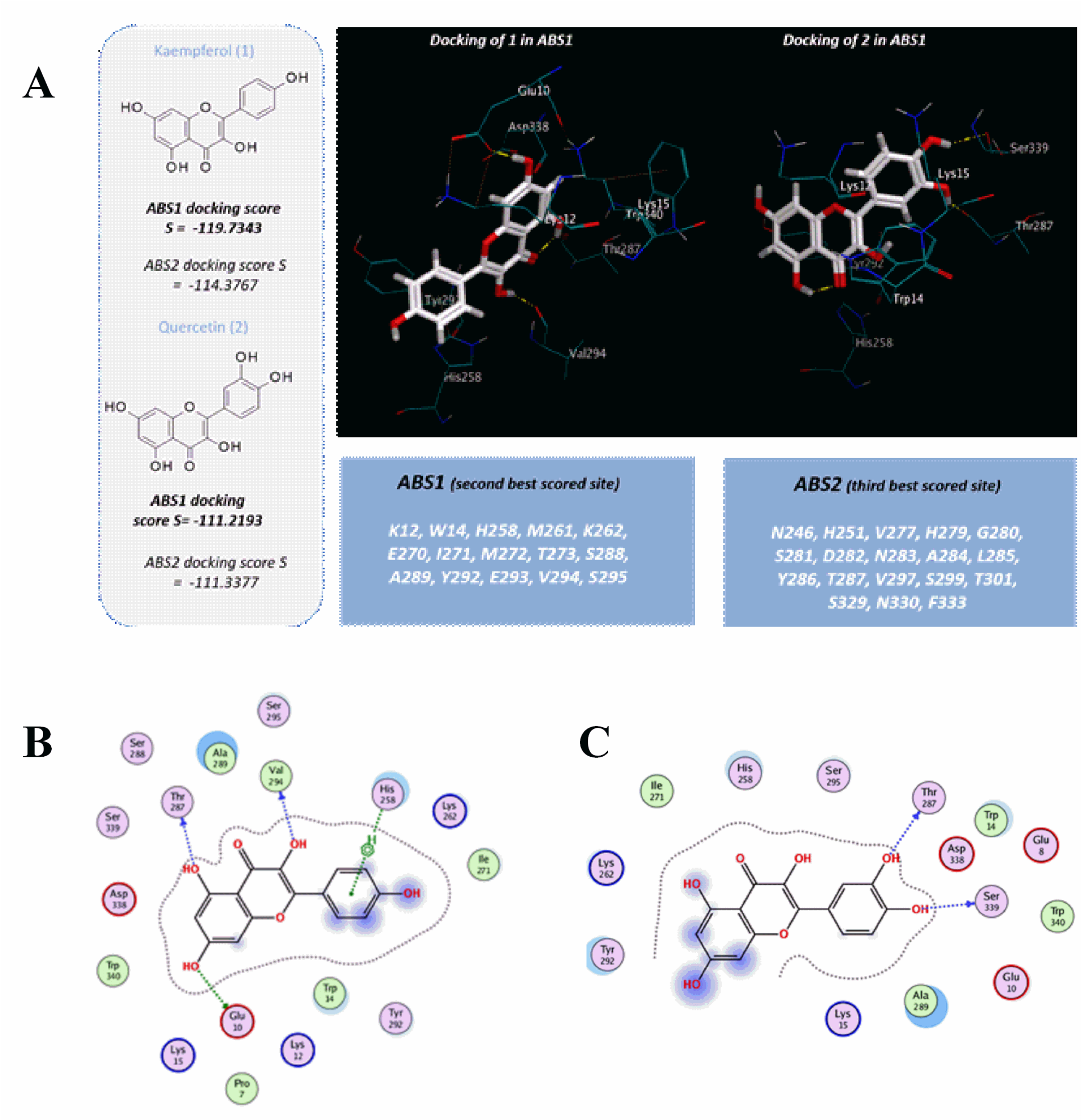

Figure S5 Docking positioning of α-glucosidase inhibitors **1** and **2** at the ABS1 binding site (**A**). Only the most relevant residues are depicted. The amino acids included in ABS1 and ABS2 are listed. Ligplot B reports the most relevant contacts between α-glucosidase and the non-competitive inhibitor **1** in ABS1. Hydrophobic and polar residues are shown in green and pink, respectively, being the negative and positive-charged amino acids detailed by red and blue circles (**B**). Ligplot C reports the most relevant contacts between α-glucosidase and the non-competitive inhibitor **2** in ABS1. Hydrophobic and polar residues are shown in green and pink, respectively, being the negative and positive-charged amino acids detailed by red and blue circles (**C**).

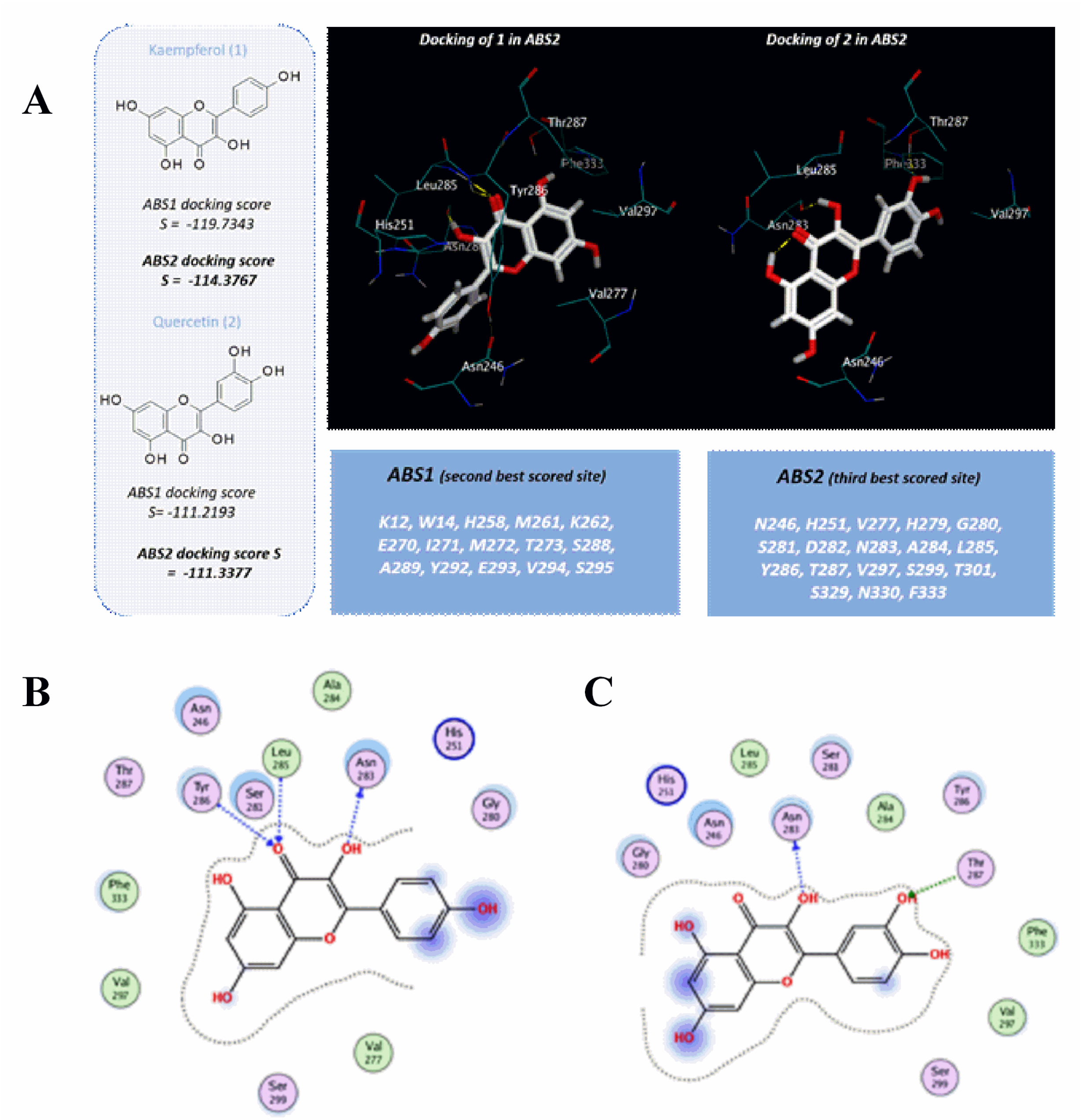

Figure S6. Docking positioning of α-glucosidase inhibitors **1** and **2** at the ABS2 binding site (**A**). Only the most relevant residues are depicted. The amino acids included in ABS1 and ABS2 are listed. Ligplot B reports the most relevant contacts between α-glucosidase and the non-competitive inhibitor **1** in ABS2. Hydrophobic and polar residues are shown in green and pink, respectively, being the negative and positive-charged amino acids detailed by red and blue circles (B). Ligplot C reports the most relevant contacts between α-glucosidase and the non-competitive inhibitor **2** in ABS2. Hydrophobic and polar residues are shown in green and pink, respectively, being the negative and positive-charged amino acids detailed by red and blue circles (**C**).

Table S1 K_m and V_{max} values of α-glucosidase with different concentrations of magnolol.

Magnolol (μM)	K_m (mM)	V_{max} (ΔOD/min)	V_{max} (μM/min) *
--	0.374 ± 0.043	0.155 ± 0.005	15.5 ± 0.50
10	0.530 ± 0.079	0.162 ± 0.008	16.2 ± 0.80
25	0.594 ± 0.080	0.148 ± 0.007	14.8 ± 0.70
50	0.538 ± 0.082	0.129 ±0.006	12.9 ± 0.60
100	0.575 ± 0.238	0.100 ± 0.013	10.0 ± 1.30

* The data were converted using the Lambert–Beer relationship.

Table S2 K_m and V_{max} of α-glucosidase with different concentrations of luteolin.

Luteolin (μM)	K_m (mM)	V_{max} (ΔOD/min)	V_{max} (μM/min) *
--	0.321 ± 0.042	0.159 ± 0.006	15.90 ± 0.60
5	0.353 ± 0.047	0.133 ± 0.006	13.30 ± 0.60
10	0.371 ± 0.055	0.119 ± 0.005	11.90 ± 0.50
25	0.357 ± 0.068	0.091 ± 0.005	9.10 ± 0.50
40	0.356 ± 0.075	0.080 ± 0.005	8.00 ± 0.50
50	0.370 ± 0.095	0.063 ± 0.006	6.34 ± 0.60

* The data were converted using the Lambert–Beer relationship.

Table S3 Km and Vmax of α-glucosidase with different concentrations of acarbose.

Acarbose (μM)	K_m (mM)	V_{max} (ΔOD/min)	V_{max} (μM/min)*
--	0.296 ± 0.080	0.132 ± 0.010	13.2 ± 1.00
200	0.908 ± 0.228	0.127 ± 0.015	12.7 ± 1.50
400	1.311 ± 0.341	0.129 ± 0.017	12.9 ± 1.70
800	1.960 ± 0.519	0.124 ± 0.020	12.4 ± 2.00
1600	3.240 ± 0.638	0.120 ± 0.016	12.0 ± 1.60
3200	5.912 ± 1.809	0.125 ± 0.030	12.5 ± 3.00
4000	8.295 ± 3.316	0.141 ± 0.047	14.1 ± 4.70

* The data were converted using the Lambert–Beer relationship.
